# Effects of artificial light at night and drought on the photosynthesis and physiological traits of two urban plants

**DOI:** 10.3389/fpls.2023.1263795

**Published:** 2023-10-12

**Authors:** Yaxi Wei, Zhen Li, Jiaolong Zhang, Dan Hu

**Affiliations:** ^1^ State Key Laboratory of Urban and Regional Ecology, Research Center for Eco-Environmental Sciences, Chinese Academy of Sciences, Beijing, China; ^2^ University of Chinese Academy of Sciences, Beijing, China; ^3^ State Key Laboratory of Simulation and Regulation of Water Cycle in River Basin, China Institute of Water Resources and Hydropower Research, Beijing, China; ^4^ Department of Water Ecology and Environment, China Institute of Water Resources and Hydropower Research, Beijing, China

**Keywords:** urban plant, artificial light at night, drought stress, fluorescence parameter, gas exchange parameter, physiological index

## Abstract

Urban plants are currently confronted with the stresses posed by artificial light at night (ALAN) and drought. A field block experiment was designed to explore the potential effects of ALAN and drought on the photosynthesis and physiological characters of two common urban plants, *Euonymus japonicus* (*E. japonicus*) and *Rosa hybrida* (*R. hybrida*). Each plant species was subjected to four distinct treatments: neither ALAN nor drought, ALAN, drought, and both ALAN and drought. The result showed the following: (1) ALAN significantly reduced the effective quantum yield (Φ_PSII_), apparent electron transfer rate (ETR), photochemical quenching parameter (_q_p), net photosynthetic (Pn), stomatal conductance (Gs), stomatal limit value (Ls), and the pigment concentrations and remarkably increased the content of malondialdehyde (MDA), total antioxidant capacity (TAC), and starch in both *E. japonicus* and *R. hybrida*. Furthermore, ALAN increased the soluble saccharides of *E. japonicus*, and this effect of ALAN also occurred on *R. hybrida* under drought. (2) Drought significantly decreased the Φ_PSII_, ETR, _q_p, Pn, Gs, Ls, and the pigment concentrations and remarkably increased the content of MDA and TAC for both *E. japonicus* and *R. hybrida*. Moreover, drought did not significantly change the starch content of both species, and it significantly increased the content of soluble saccharides for *E. japonicus.* (3) The interaction between ALAN and drought occurred on the Φ_PSII_, ETR, Pn, MDA, and TAC of *E. japonicus*, but had no effect on *R. hybrida*. For urban areas affected by ALAN and drought, it is advisable to select plant species with strong stress resistance for gardening purposes, and plants directly exposed to ALAN should receive sufficient water during hot and dry weather conditions to maintain their normal growth.

## 1 Introduction

Since the early 20th century, due to the extensive use of artificial light at night (ALAN), the natural patterns of day and night that both play an important role in growth, production, and reproduction of living organisms and maintaining ecosystem health and services have been disrupted, resulting in light pollution (Ayalon et al., 2019; Falchi et al., 2019; Sanders et al., 2021). Recent reports indicate that more than 20% of the global land surface is subject to light pollution, and currently, areas exposed to such pollution are increasing at a growing rate (Falchi et al., 2016; Ffrench-Constant et al., 2016; Falchi et al., 2019; Sanders et al., 2021). Furthermore, the increasing prevalence of nighttime light, with its diverse spectrum, intensity, and duration of exposure, has raised considerable concerns regarding its direct and indirect ecological impacts (Salmon and Witherington, 1995; Firebaugh and Haynes, 2019; Levy et al., 2020). A series of influence of ALAN on foraging, pollination, mating, and other behaviors of a wide variety of animals and phenology of plants have been well documented in recent years (Salmon and Witherington, 1995; Ffrench-Constant et al., 2016; Firebaugh and Haynes, 2019; Lian et al., 2021).

Currently, the effects of ALAN on plant physiology have increasingly attracted much attention from researchers (Poulin et al., 2014; Bennie et al., 2016; Hey et al., 2020). Czaja and Kolton (2022) and Ffrench-Constant et al. (2016) found that ALAN can advance the timing of budburst in trees in the spring. Skvareninova et al. (2017) showed that ALAN can delay the fall phenology of trees. The change of phenology is inevitably accompanied by physiological changes. Czaja and Kolton (2022) pointed out that the advance of spring phenology is related to soluble sugar, but the mechanism of the influence of ALAN on plant physiology is still unclear. As an energy source, ALAN may have a limited effect on net photosynthesis in plants due to its relatively low quantum flux densities compared to sunlight, but theoretically, ALAN has the potential to trigger slight photosynthesis in certain plants, particularly in cave systems (Gaston et al., 2013; Poulin et al., 2014). Additionally, as a source of information, ALAN perceived by photoreceptors can disrupt the normal transmission of information, leading to misleading signals and disturbances in the signaling and functions of photoreceptors (Gaston et al., 2013), which can change its function in the daytime. These changes can reshape the structure and function of ecosystem to some extent (Bennie et al., 2016; Knop et al., 2017; Gaston and Holt, 2018). Although the threshold of light intensity at night required to cause significant physiological impacts is unknown (Gaston et al., 2013), some obvious changes in plants have been identified under ALAN within levels of anthropogenic light pollution. Lo Piccolo et al. (2023) have shown that the CO_2_ assimilation rate decrease in *Platanus × acerifolia* and *Tilia platyphyllos* under ALAN. Zhang et al. (2020) provided evidence that as the hours of lighting increase at night, the increased malondialdehyde concentration (MDA) and decreased value of the apparent electron transfer rate (ETR) were observed in *Lolium perenne*. However, the light intensity used in these experiments was higher than light compensation points of the plants. In fact, except for the parts of plants near the light source, the intensity of ALAN received by plants is typically below the compensation point. Little is known about the effects of ALAN with the intensity below light compensation point on plant photosynthesis and physiological characteristics.

Urban ecosystem, being heavily impacted by human activities, is not only highly exposed to ALAN but also susceptible to drought due to the urban heat island effect. Many studies have confirmed that drought could have a series of negative effects on urban plants, including the reduction of leaf pigment, photosynthetic rate, and biomass (Xu et al., 2010; Singh and Raja Reddy, 2011; Wang et al., 2019; Talbi et al., 2020). However, there have been no investigations into the combined effects of ALAN and drought on plant physiology and ecology. There is a need to take ALAN especially with light intensity below the light compensation point into consideration when investigating the effects of the drought (Bennie et al., 2016; Davies and Smyth, 2018) to ensure the health and sustainable ecological function of urban ecosystem under the trend of those environmental stresses increasing in the future.

The objective of our study was to investigate the effects of ALAN and drought on the photosynthesis and physiological characters of urban garden plants. *Euonymus japonicus* (*E. japonicus*) and *Rosa hybrida* (*R. hybrida*), which are frequently utilized for urban greening purposes in Beijing, were chosen as our model organisms. Based on the photosynthetic parameters and physiological traits of the two species, we assumed that ALAN and drought negatively impact plant growth, and the interaction of ALAN and drought was remarkable on some indicators of the plant.

## 2 Materials and methods

### 2.1 Experimental design

A field manipulation experiment was conducted in Yanqing (40° 29′ N, 115°59′ E), northwest of Beijing, China. In April 2022, an experimental area, measuring 24 × 6 m was selected and divided into six experimental plots. Each plot was 2 × 2 m and there was a 2-m margin between plots. Lit treatments consisting of a white LED (correlated color temperature, 6500K), which was mounted 1.7 m above the ground on a wooden frame, were added to half of the six field plots. The mean light intensity measured by an illuminometer (XI MA, China) on the ground of the plots was 123 lux. During the measurement, the light intensity received by the uppermost part of *E. japonicus and R. hybrida* were approximately 385 lux and 443 lux, which are close to 8 μmol photons m^−2^ s^−1^ and 9 μmol photons m^−2^ s^−1^, respectively, and below their light compensation points (You et al., 2014; Zhang et al., 2014). The irradiation time of LED lamps was controlled by a light-sensitive switch that turned on the lamps at dusk and turned off lamps at dawn. In the rest of the plots, no lighting equipment was installed, so they were free from light at night. During the whole experiment, we turned off the surrounding light sources and used lampshades and black plastic plates to shade the light source that was mounted on wooden frame, so the illumination of the surrounding and non-ALAN plots was close to zero on the nights without moonlight.

Subsequently, 2-year-old *E. japonicus* and *R. hybrida* seedlings were obtained from a nursery at Yongchun station, Beijing, China in April 2022. *E. japonicus* and *R. hybrida* samplings with a mean height of 76.46 cm and 62.81 cm, respectively, and a basal diameter of 15.68 mm and 14.42 mm, respectively, were transplanted to seedling trays (length × width × height, 28 cm × 22 cm × 31 cm), at one seedling per pot. Each pot contained approximately 4 kg of soil consisting of field topsoil and flower soil. For the soil mix, the bulk density was 1.21 g/cm^3^, total carbon was 37.90 g/kg, total nitrogen was 2.03 g/kg, available phosphorus was 8.12 mg/kg, available potassium was 75.12 mg/kg, and the pH value was 7.36. To avoid nutrient deficiency affecting plant growth, we added 10 g of slow-release fertilizer to each pot. Before the drought control test, all pots were given enough water to ensure the healthy growth of the plants. Finally, 36 pots of *E. japonicus* and 36 pots of *R. hybrida* were randomly divided equally into six plots.

After a period of 50 days, the level of drought stress was determined referring the method described by Gao et al. (2017). The *E. japonicus* and *R. hybrida* in each plot were divided into two groups, respectively. Two irrigation treatments were applied during the experiment. Every 2 days, one group was well irrigated to keep the field capacity, and the other group was irrigated with 40% of the water used in the first group. Three pots were randomly selected from the no drought and drought treatments, respectively, and soil water content was recorded at 15 cm of the pot with a datalogger (EC-5, USA) on the day when no watering was performed. For the whole experimental period, the average soil water contents of no drought and drought conditions were 19.20% ± 1.58% and 12.33% ± 1.39% respectively. Watering and soil water content recording were both performed between 6:00 and 7:30 p.m. When it rained, we covered all pots with a plastic cover. Briefly, there were four treatments: No ALAN + No drought, ALAN + No drought, No ALAN + drought, and ALAN + drought. For each treatment and species, there were three plots as replicas, and each plot had three pots. (Field test images are shown in **Figure S1**).

### 2.2 Measurements of chlorophyll fluorescence and gas exchange parameters

In early August 2022, two sunny windless days without watering were selected for the measurement of *E. japonicus* and *R. hybrida*, respectively. For each species in each plot with different treatments, two fully expanded leaves facing the sun from two different plants were selected for measurements from 8:30 to 11:30, using a portable photosynthesis system (LI-6400XT, LI-CORCOR, USA) equipped with a fluorescent leaf chamber. During the measurements, photosynthetic active radiation was 1,200 μmol m^−2^ s^−1^, the gas flow rate was 500 mmol s^−1^, the relative moisture was 44%–56%, CO_2_ concentration was ambient and was provided by a buffer bottle, and the leaf chamber temperatures measured for *E. japonicus* and *R. hybrida* were 32.05 ± 0.73°C and 31.77 ± 0.63°C, respectively. The following parameters were obtained in the measurement: (1) chlorophyll fluorescence parameters: effective quantum yield (Φ_PSII_), ETR, and photochemical quenching parameter (_q_p); and (2) gas exchange parameters: net photosynthetic rate (Pn, μmolCO_2_ m^−2^ s^−1^), stomatal conductance (Gs, molH_2_O m^−2^ s^−1^), and stomatal limit value (Ls).

### 2.3 Measurements of photosynthetic pigments

After chlorophyll fluorescence and gas exchange parameters were measured, leaf samples were collected using a hole punch. Chlorophyll and carotenoid were extracted in 5 mL of 95% ethanol at 4°C for 72 h under dark conditions and their concentrations were measured at 470, 649, and 665 nm by a UV spectrophotometer (Agilent, USA), and calculated using the equations proposed by Lichtenthaler and Wellburn (1982).

### 2.4 Measurements of MDA, total antioxidant capacity, soluble saccharides, and starch

For each species, two plants were selected from each plot with different treatments and 10 healthy and fully spread leaves on the top were collected from each plant after measuring photosynthesis parameters. The collected leaves were divided into fresh and dry samples. Fresh samples were immediately frozen in liquid nitrogen and stored in a freezer at −80°C for the determination of MDA and total antioxidant capacity (TAC). Dry samples were dried in an oven 70°C until the dry weight stabilized for the determination of soluble saccharides and starch content.

MDA was measured according to the method of Heath and Packer (1965) with appropriate modification. Approximately 0.07 g of fresh-frozen leaf tissue was homogenized with 4 mL of 10% trichloroacetic acid. After the homogenate was centrifuged at 12,000×*g* for 3 min at 4°C, 2 mL of supernatant and 2 mL of 0.6% thiobarbituric acid were mixed and heated in a boiling water bath at 100°C for 15 min and then the mixture was quickly cooled in an ice bucket to stop the reaction. Then, samples were centrifuged at 12,000×*g* for 3 min to collect the supernatants that were measured at 450 nm, 532 nm, and 600 nm by a UV spectrophotometer.

TAC was measured according to the method of Benzie and Strain (1996) with appropriate modification. Approximately 0.06 g of fresh-frozen leaf tissue was homogenized with 4 mL of 70% ethanol. After the homogenate was centrifuged at 12,000×*g* for 3 min at 4°C, 100 μL (dilute the extract if necessary) of supernatant and 5 mL of FRAP reagent were mixed and reacted at 37°C for 10 min. Then, samples were centrifuged at 12,000×*g* for 3 min to collect the supernatants that were measured at 593 nm by a UV spectrophotometer. The total antioxidant capacity of the sample was expressed as equivalent to the reducing power of Fe^2+^.

Soluble saccharides and starch were measured according to the method of Fairbairn (1953) and Clegg (1956) with appropriate modification. Approximately 0.1 g of dried leaf tissue was homogenized with 4 mL of 80% ethanol, and the homogenate was heated in a boiling water bath at 80°C for 40 min. The reaction mixture was centrifuged at 12,000×*g* for 3 min to collect the supernatants. The extraction process was repeated and the supernatant was incorporated. Then, 10 mg of activated carbon was added into the supernatant and decolorized at 80°C for 30 min. After filtration, 100 μL (dilute the extract if necessary) of supernatant and 5 mL of anthrone reagent were mixed, and then the mixed solution was measured at 625 nm by a UV spectrophotometer. The residue after the extraction of soluble saccharides was transferred to a volumetric bottle with 5 mL of water, and heated in a boiling water bath at 100°C for 15 min. Then, 2 ml of 9.2 mol/L perchloric acid was added to the volumetric bottle, and heating was continued for 5 min at 100°C. The reaction mixture was centrifuged at 12,000×*g* for 3 min to collect the supernatants. The extraction process was repeated and the supernatant was recovered. The content of starch was determined by the anthrone chromogenic method.

### 2.5 Statistical analysis

A two-way analysis of variance followed by the Fisher’s least significant difference (LSD) was used for analyzing the main effects of ALAN, drought, and their interaction on photosynthetic and physiological parameters. Before statistical analysis, the homogeneity of variance and normality of distribution of data were tested. All statistical analyses were performed using IBM SPSS Statistics Version 22 software package, and the graphs were drawn with Origin 2023 software.

## 3 Results

### 3.1 Fluorescence parameters and gas exchange parameters

**Figure 1:**
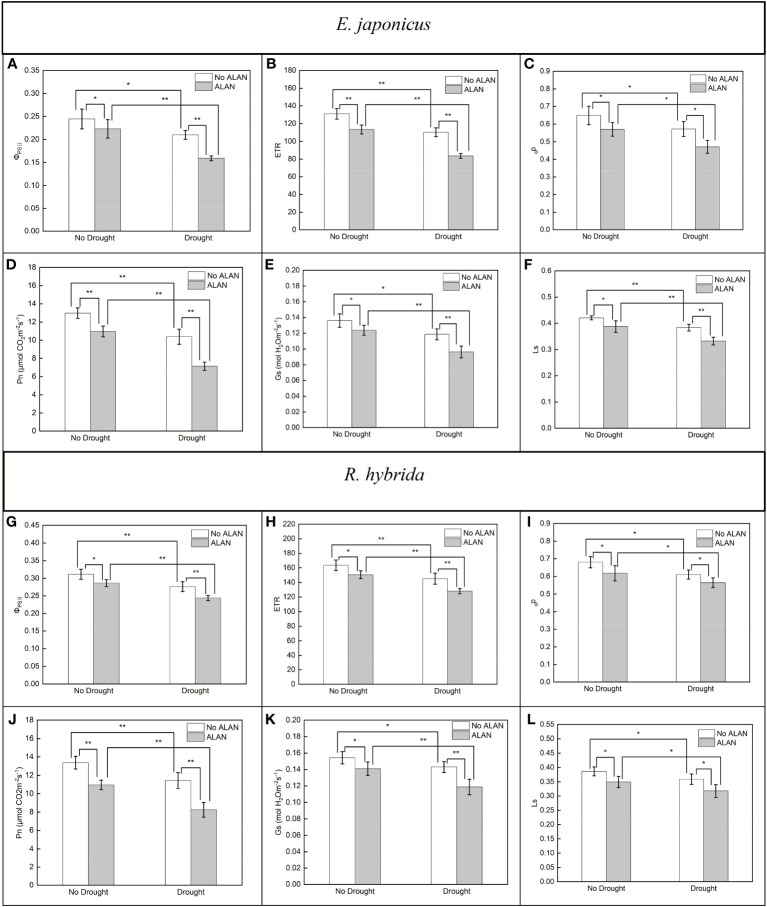
Effects of ALAN and drought on Φ_PSII_, ETR, _q_p, Pn, Gs, and Ls of *E. japonicus* **(A–F)** and *R. hybrida* **(G–L)**. Bars are means± SDs. *Significant at *p* < 0.05 and **Significant at *p* < 0.001.

ALAN significantly decreased the values of Φ_PSII_, ETR, _q_p, Pn, Gs, and Ls of *E. japonicus* and *R. hybrida* under no drought and drought conditions (**Figure 1**). Similarly, drought also significantly decreased these indicators of the two species under no ALAN and ALAN conditions (**Figure 1**). Furthermore, the values of Φ_PSII_, ETR, and Pn of *E. japonicus* were significantly affected by the interaction of ALAN and drought (**Table 1**). The mean effects of ALAN on the values of Φ_PSII_, ETR, and Pn were greater under drought (24.18%, 24.14%, and 31.27%, respectively) than those under no drought (8.78%, 13.40%, and 15.53%, respectively). Also, the mean effects of drought on the values of Φ_PSII_, ETR, and Pn were greater under ALAN (28.63%, 26.24%, and 34.89%, respectively) than those under no ALAN (14.13%, 15.80%, and 19.98%, respectively) (**Figures 1A, B, D**). However, the interaction of ALAN and drought did not significantly affect the fluorescence parameters and gas exchange parameters of *R. hybrida* (**Table 1**).

**Table 1:** Test of between-subjects effects of ALAN and drought on chlorophyll fluorescence and gas exchange parameters of *E. japonicus* and *R. hybrida*.

Species	Source of variation	Φ_PSII_	ETR	_q_p	Pn	Gs	Ls
*E. japonicus*	ALAN	**	**	**	**	**	**
	Drought	**	**	**	**	**	**
	ALAN*Drought	*	*	ns	*	ns	ns
*R. hybrida*	ALAN	**	**	*	**	**	**
	Drought	**	**	**	**	**	*
	ALAN*Drought	ns	ns	ns	ns	ns	ns

*Significant at p < 0.05 and **Significant at p < 0.001.

ns, no significant.

### 3.2 Photosynthetic pigments

**Figure 2:**
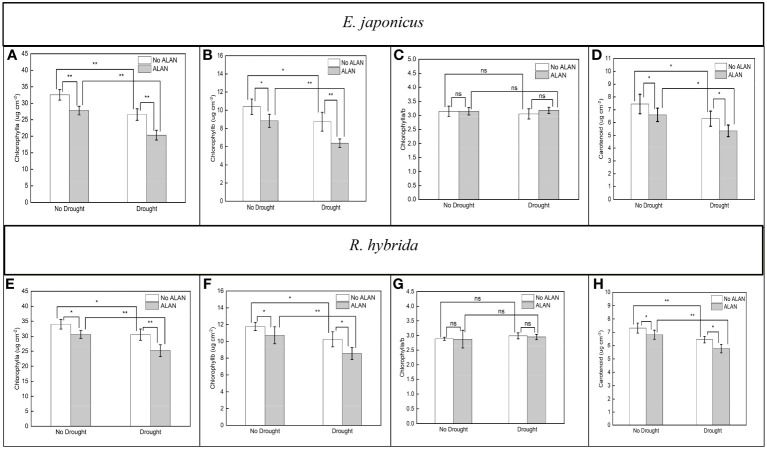
Effects of ALAN and drought on Chlorophyll a, Chlorophyll b, Chlorophyll a/b, and carotenoid of *E. japonicus* **(A–D)** and *R. hybrida* **(E–H)**. Bars are means± SDs. *Significant at *p* < 0.05 and **Significant at *p* < 0.001. ns, no significant.

ALAN significantly reduced the contents of Chlorophyll a, Chlorophyll b, and carotenoid, and did not significantly affect the Chlorophyll a/b of *E. japonicus* and *R. hybrida* under no drought and drought (**Figure 2**). Similarly, drought also significantly reduced the pigment contents and did not affect the Chlorophyll a/b of the two species under no ALAN and ALAN (**Figure 2**). However, the interaction of ALAN and drought did not significantly change the above indicators of both species (**Table 2**).

**Table 2:** Test of between-subjects effects of ALAN and drought on pigment contents of *E. japonicus* and *R. hybrida*.

Species	Source of variation	Chlorophyll a	Chlorophyll b	Chlorophyll a/b	Carotenoid
*E. japonicus*	ALAN	**	**	ns	*
	Drought	**	**	ns	**
	ALAN*Drought	ns	ns	ns	ns
*R. hybrida*	ALAN	**	**	ns	**
	Drought	**	**	ns	**
	ALAN*Drought	ns	ns	ns	ns

*Significant at p < 0.05 and **Significant at p < 0.001.

ns, no significant.

### 3.3 MDA and TAC

**Figure 3:**
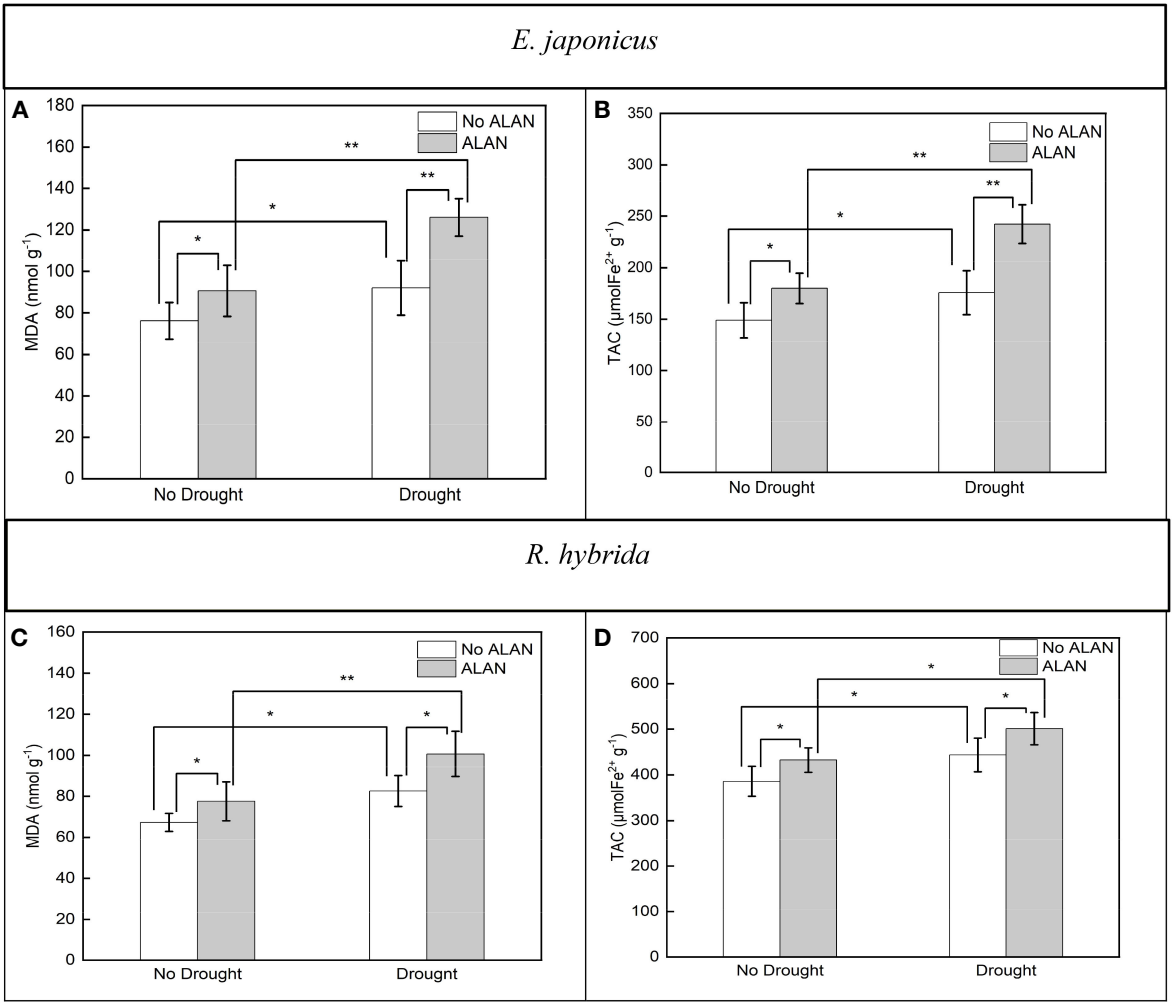
Effects of ALAN and drought on MDA and TAC of *E. japonicus* **(A, B)** and *R. hybrida* **(C, D)**. Bars are means± SDs. *Significant at *p* < 0.05 and **Significant at *p* < 0.001.

ALAN significantly increased the contents of MDA and TAC of *E. japonicus* and *R. hybrida* under no drought and drought (**Figure 3**). Similarly, drought also significantly increased these indicators of the two species under no ALAN and ALAN (**Figure 3**). MDA and TAC of *E. japonicus* were significantly affected by the interaction between ALAN and drought (**Table 3**). Under no drought, ALAN increased MDA and TAC by 19.03% and 20.91%, respectively, whereas under drought, ALAN increased MDA and TAC by 36.94% and 37.92%, respectively. Similarly, under no ALAN, drought increased MDA and TAC by 20.85% and 18.00%, respectively, whereas under ALAN, drought increased MDA and TAC by 39.04% and 34.61%, respectively (**Figures 3A, B**). However, the interaction of ALAN and drought did not significantly affect MDA and TAC of *R. hybrida* (**Table 3**).

**Table 3:** Test of between-subjects effects of ALAN and drought on MDA and TAC of *E. japonicus* and *R. hybrida*.

Species	Source of variation	MDA	TCA
*E. japonicus*	ALAN	**	**
	Drought	**	**
	ALAN*Drought	*	*
*R. hybrida*	ALAN	*	*
	Drought	**	**
	ALAN*Drought	ns	ns

*Significant at p < 0.05 and **Significant at p < 0.001.

ns, no significant.

### 3.4 Soluble saccharides and starch

**Figure 4:**
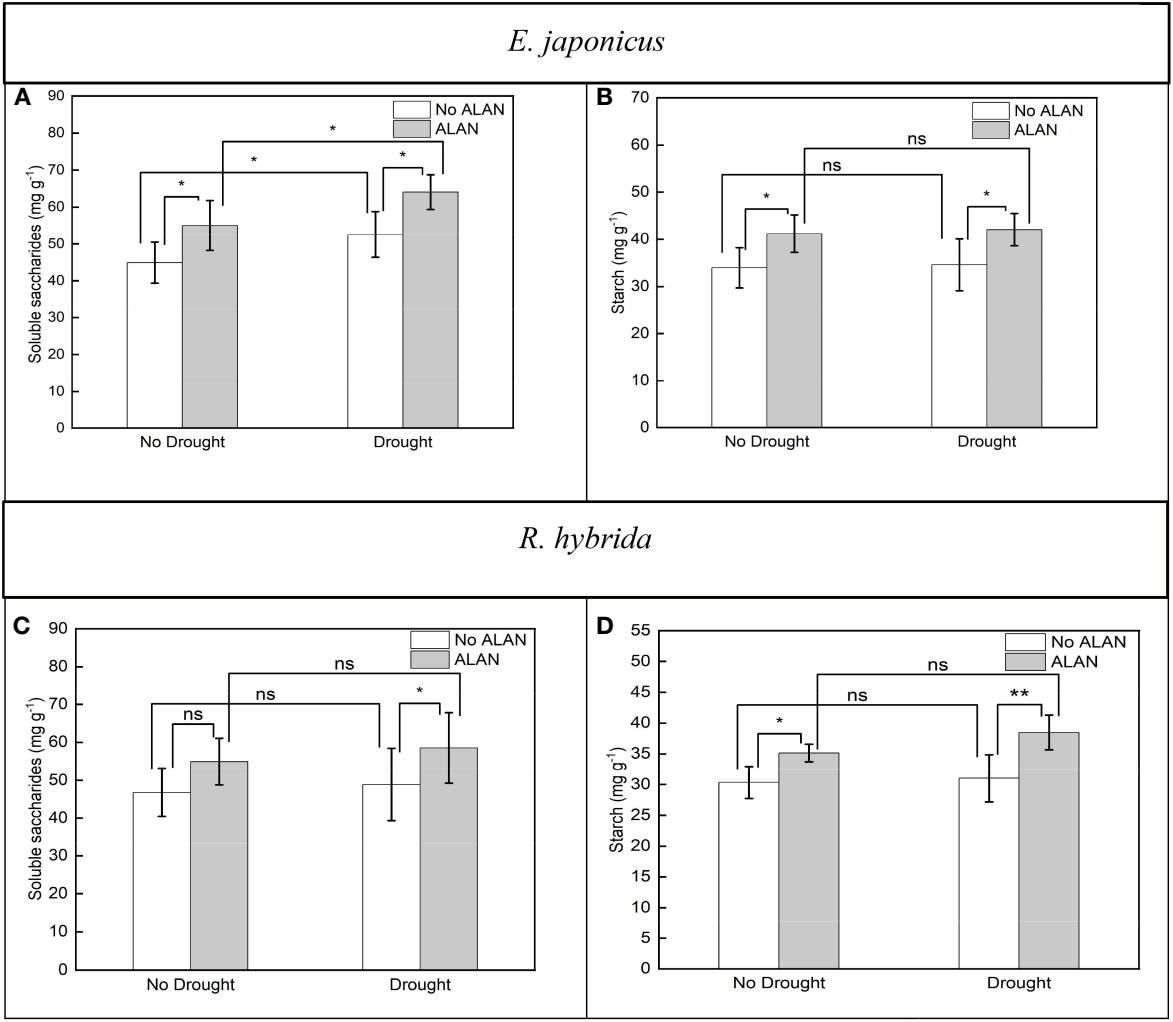
Effects of ALAN and drought on soluble saccharides and starch of *E. japonicus* **(A, B)** and *R. hybrida* **(C, D)**. Bars are means± SDs. *Significant at *p* < 0.05 and **Significant at *p* < 0.001. ns, no significant.

For *E. japonicus*, ALAN significantly increased the soluble saccharides content under no drought and drought. Similarly, drought significantly increased the soluble saccharides contents under no ALAN and ALAN (**Figure 4A**). For *R. hybrida*, ALAN significantly increased the soluble saccharides content under drought conditions but did not significantly impact it under no drought conditions (**Figure 4C**), and drought had no significant effect on the soluble saccharides content (**Table 4**). For both species, ALAN significantly increased the starch contents under no drought and drought, and drought did not significantly change the starch contents under no ALAN and ALAN (**Figures 4B, D**). Furthermore, the interaction of ALAN and drought did not significantly changet he soluble saccharides and starch content of the two species (**Table 4**).

**Table 4:** Test of between-subjects effects of ALAN and drought on soluble saccharides and starch of *E. japonicus* and *R. hybrida*.

Species	Source of variation	Soluble saccharides	Starch
*E. japonicus*	ALAN	**	*
	Drought	*	ns
	ALAN*Drought	ns	ns
*R. hybrida*	ALAN	*	**
	Drought	ns	ns
	ALAN*Drought	ns	ns

*Significant at p < 0.05 and **Significant at p < 0.001.

ns, no significant.

## 4 Discussion

Urban plants are stressed by both ALAN and drought due to the use of artificial light sources and urban heat islands. Under stress, plants will change their appearance, morphology structure, and biochemical metabolic activities in response to stress (Gilbert and Medina, 2016). When the photosynthetic system is faced with adverse environmental conditions, the activity of its photosystem II (PSII) will be inhibited, known as photoinhibition (Aro et al., 1993; Murata et al., 2007). After exposure to ALAN and drought stresses, the values of Φ_PSII_, ETR, and _q_p in *E. japonicus* and *R. hybrida* decreased significantly, indicating that PSII was impaired and the ability of light capture and electron transfer in their leaves decreased. Fluorescence characteristics of chlorophyll molecules are crucial for understanding the energy transfer efficiency in chloroplasts, and fluorescence measurement is a non-destructive measurement of studying photosynthesis efficiency (Ball et al., 1994). Φ_PSII_ stands for the proportion of absorbed light energy utilized in photochemical reactions to the total absorbed light energy and ETR provides an empirical estimation of the rate of electron flow through the electron transport chain (Park and Dinh, 2019). The photosynthetic system of the plant is very sensitive to light at night and it was well documented that an extremely low light intensity would cause marked changes in plant physiological and behavior processes (Raven and Cockell, 2006; Poulin et al., 2014). Darkness during the diurnal cycle is essential for plant repair and recovery from adverse environmental conditions, as it serves as an important resource for certain physiological processes in plants (Gaston et al., 2013; Singhal et al., 2019). However, ALAN replaces the dark phase of the diurnal cycle, creating an environment where the natural nighttime brightness is altered. For plants, changing the light pattern would bring pressure to the circadian rhythm and produce reactive oxygen species (ROS) (**Table S1** and **Figure S2**), which suppressed the repair of photodamaged PSII by inhibiting the synthesis of proteins (Murata et al., 2007; Velez-Ramirez et al., 2011). Similarly, many studies have shown that drought, a decline in water, reduces electron transport and the production of ATP and NADPH, inducing the generation of ROS (Wang et al., 2019; Shin et al., 2021), which is consistent with our findings (**Table S1** and **Figure S2**). The value of _q_p characterizes the energy expenditure used for photosynthesis and its decrease in our study illustrated that the absorbed light dissipated more through a thermal reaction and not by using a photochemical reaction under ALAN and drought (Wang et al., 2019). In our study, the significant decrease in Φ_PSII_, ETR, and _q_p caused by photoinhibition provided a possible explanation for the decreased Pn.

The reduction of photosynthetic rate under an adverse environment is mainly attributed to stomatal factors linked to stomatal closure and non-stomatal factors associated with photoinhibition (Xu et al., 2010; Wang et al., 2019; Talbi et al., 2020). When the photosynthesis rate declines, if the Ls increased, the photosynthetic rate was mainly controlled by stomatal regulation; if the Ls decreased, the decrease of photosynthetic rate was mainly attributed to non-stomatal regulation (Wang et al., 2019). Under ALAN and drought stress, the Pn and Ls of *E. japonicus* and *R. hybrida* decreased significantly, which was consistent with the non-stomatal limitation. ALAN and drought can restrain electron transport, the synthesis of ATP and NADPH, and the catalysis of Rubisco, resulting in excessive ROS, which, in turn, increases damage to PSII and suppresses the repair of PSII (Allakhverdiev et al., 2005; Murata et al., 2007; Wang et al., 2019). However, we also found that Gs decreased under ALAN and drought conditions, indicating that stomatal factors also inhibit photosynthesis to a certain extent. Plants exposed to ALAN could show a decreased stomatal width, length, and size (Kwak et al., 2017), and the changed stomatal parameters can reduce the stomatal conductance of plants. When plants encounter drought, they will reduce stomatal conductance to prevent excessive water loss. The decrease of Gs under ALAN and drought can limit CO_2_ availability for photosynthesis, leading to a reduction in NADPH and a lower Pn (Xu et al., 2010; Wang et al., 2019).

In our research, the pigment content of *E. japonicus* and *R. hybrida* decreased significantly under ALAN and drought stresses, which was similar to some previous related studies (Guerfel et al., 2009; Haque et al., 2015; Dias et al., 2018; Zhang et al., 2020). Generally, the pigment content reduction under ALAN and drought is due to photooxidation, which can lead to degradation of the pigment and chlorophyll synthesis deficiency (Singh and Raja Reddy, 2011; Dias et al., 2018). In addition, decreased pigment content is known as a pressure response mechanism to reduce light absorption by chloroplasts (Zhang et al., 2019). Change of Chla/b ratio could affect light-adapted photosynthetic apparatus, the capacity for electron transport, and Calvin cycle enzymes (Terfa et al., 2013). The results of this study showed that ALAN and drought did not significantly affect the Chlorophyll a/b of *E. japonicus* and *R. hybrida*, suggesting that these two plants have a photoprotective mechanism to ensure the stability of the photosynthetic system under ALAN and drought stresses.

In this experiment, there was a significant increase in MDA for both *E. japonicus* and *R. hybrida* under ALAN and drought conditions, indicating that the plants were stressed by ALAN and drought. Considering photoinhibition, ALAN and drought could disrupt the balance of light energy capture and light energy utilization and cause excessive ROS, resulting in peroxidation of membrane lipid. MDA is the final decomposition product of membrane lipid peroxidation, and its content links to the degree of injury suffered by plants under adverse conditions (Zhang et al., 2020). It is evident that the increasing MDA has been found in some species under continuous light (Bian et al., 2018; Zhang et al., 2020). Also, drought can cause oxidative stress characterized by the increased MDA that had been well documented in many studies (Singh and Raja Reddy, 2011; Talbi et al., 2020). Normally, the content of ROS in plant cells is in a state of dynamic balance (Zhang et al., 2019), and when plants are subjected to stress, they usually improve their antioxidant capacity to reduce ROS and prevent cell damage. In this research, the TAC of the two species significantly increased under ALAN and drought, which indicated that the excessive ROS production in ALAN and drought conditions stimulated the antioxidant machinery in the plants (Levy et al., 2020). However, this compensatory response was not enough to offset the high formation of lipid peroxidation (Levy et al., 2020), leading to a higher MDA finally.

Soluble saccharides and starch are the primary forms of non-structural carbohydrates (NSCs) stored in plants, and they play a crucial role in various physiological functions, including metabolism, osmotic regulation, defense mechanisms, water transport, and embolic repair (Marquis et al., 1997; Sakamaki and Ino, 2004; Resco de Dios and Gessler, 2021). The observed increase in soluble saccharides and starch content under ALAN in our study might be responsible for the downregulation of Pn (Haque et al., 2015; Haque et al., 2017). During the day, plants carry out photosynthesis, fix CO_2_, and accumulate NSCs, and the accumulated NSCs can be used to support plant metabolism at night (Velez-Ramirez et al., 2011). Continuous light exposure can inhibit or downregulate starch-degrading enzymes, which can reduce export of carbohydrates from source leaves to sinks during the night, leading to their excessive accumulation in leaves (Haque et al., 2015). As previously discussed, accumulation of carbohydrates could lower the amount and activity of Rubisco, reduce electron acceptors and generate more ROS, and these changes in turn caused oxidative damage and downregulated photosynthesis (Velez-Ramirez et al., 2011; Haque et al., 2015). Moreover, the excessive accumulation of sediment in the chloroplast could damage the chloroplast structure, increase the length of CO_2_ diffusion pathway in the chloroplast, and adversely affect the photosynthesis in plants (Kwak et al., 2017). We also found that drought significantly increased the soluble saccharides of *E. japonicus*. As the main osmotic regulator of plant stress, the content of soluble saccharides will increase rapidly to maintain turgor pressure under drought stress to increase the ability of plant to resist drought stress (Guo et al., 2021; Resco de Dios and Gessler, 2021). However, drought did not significantly change soluble saccharides of *R. hybrida*, which suggested that the effects of drought on soluble saccharides vary with species. Furthermore, drought did not significantly affect the content of starch in *E. japonicus* and *R. hybrida*, which aligns with previous studies (Gruber et al., 2012). The response of starch to drought stress in plants remains inconclusive, likely due to variations in plant species and the intensity and duration of drought (Guo et al., 2021). It is important to note that the diurnal variation in soluble saccharides and starch content should be considered when studying the influence of stresses on leaf carbohydrate metabolism (Haque et al., 2015). The time of day at which carbohydrate measurements are taken can significantly impact the results. Further research is needed to explore how carbohydrates change at different times of the day under the effects of ALAN and drought stresses.

In our study, the values of Φ_PSII_, ETR, and Pn and the contents of MDA and TAC in *E. japonicus* showed a significant interaction between ALAN and drought. Hey et al. (2020) revealed that ALAN could interact with soil moisture to affect plant growth, which might be because ALAN inhibited plant proper stomatal functioning and the corresponding improper stomatal functioning decreased water use efficiency and carbon assimilation efficiency. Although, in our study, the decrease in the photosynthetic rate under combined ALAN and drought conditions was mainly attributed to nonstomatal regulation, the stomatal regulation also affected the photosynthetic growth to some extent, which agreed with previous studies (Kwak et al., 2017). In our study, both ALAN and drought lead to a high level of MDA, and once the accumulation of MDA exceeds a certain threshold, the interaction of ALAN and drought may strongly affect the growth of plants. However, no significant interaction was found in *R. hybrida*, which may be due to the fact that *R. hybrida* has stronger antioxidant capacity to remove ROS. It remains to be determined that if the intensity of the ALAN or the drought increases, the interaction of ALAN and drought would significantly affect *R. hybrida*.

In our study, we only used one light intensity, one spectrum, and one drought level. The interaction of ALAN and drought on plants is complex and it may vary with light intensity, spectral composition, drought degree, and plant species. Future studies should elucidate the physiological mechanisms of light and drought interactions in more plant species with various nighttime light environments and drought levels. For urban areas affected by ALAN and drought, it is advisable to select plant species with strong stress resistance for gardening purposes, and plants directly exposed to ALAN should receive sufficient water during hot and dry weather conditions to maintain their normal growth.

## 5 Conclusion

Findings here show that ALAN and drought could cause photoinhibition and oxidative stress, which produced disturbances in photosynthetic machinery and led to the decrease in photosynthetic parameters (Φ_PSII_, ETR, _q_p, Pn, and Ls) and pigment contents and the increase in MDA and TAC contents for both species. Furthermore, ALAN and drought can also decrease the Gs of both species, which can also affect the photosynthetic capacity. ALAN inhibited soluble saccharides and starch export from the source leaves to sinks at night, resulting in the accumulation of non-structural carbohydrates, which may cause oxidative damage, destroy the chloroplast structure, and lower the photosynthesis. Drought did not significantly change the starch content for both plants, and it significantly increased the content of soluble saccharides of *E. japonicus*. In addition, the interaction of ALAN and drought had a significant effect on Φ_PSII_, ETR, and Pn and the contents of MDA and TAC in *E. japonicus*, but had no significant effect on all indexes of *R. hybrida*. Future research should endeavor to elucidate the physiological mechanisms underlying the interactions between ALAN and drought stress in a broader spectrum of plant species. This exploration should encompass various nocturnal light conditions and levels of drought severity. In the context of urban areas impacted by both ALAN and drought stress, a judicious selection of garden vegetation should prioritize species exhibiting robust stress resistance. Furthermore, for plant species subjected to direct ALAN, increased irrigation during periods of elevated temperatures and arid conditions becomes imperative to sustain their optimal developmental trajectory.

## Data availability statement

The raw data supporting the conclusions of this article will be made available by the authors, without undue reservation.

## Author contributions

YW: Formal Analysis, Investigation, Methodology, Software, Writing – original draft. ZL: Investigation, Writing – review & editing. JZ: Investigation, Writing – review & editing. DH: Funding acquisition, Writing – review & editing.
