# Supplementary material for: Effects of artificial light at night and drought on the photosynthesis and physiological traits of two urban plants
Source: Front Plant Sci. 2023 Oct 12;14:1263795. doi: 10.3389/fpls.2023.1263795 (PMC10602676; doi:10.3389/fpls.2023.1263795)
Supplement: Supplementary file 1 [file DataSheet_1.docx]

Supplementary Materials

| 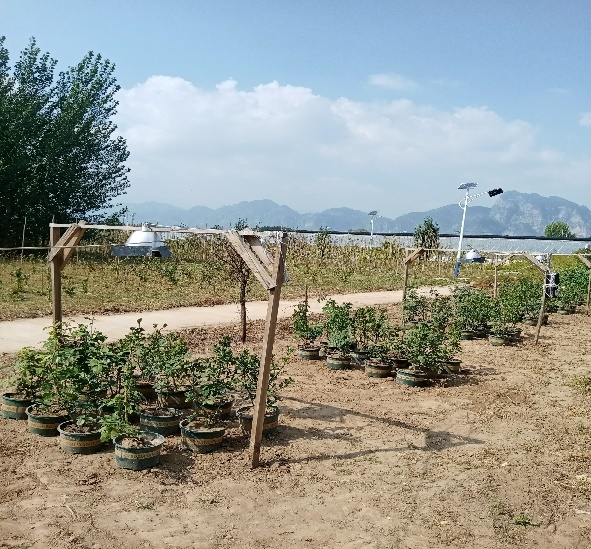 | 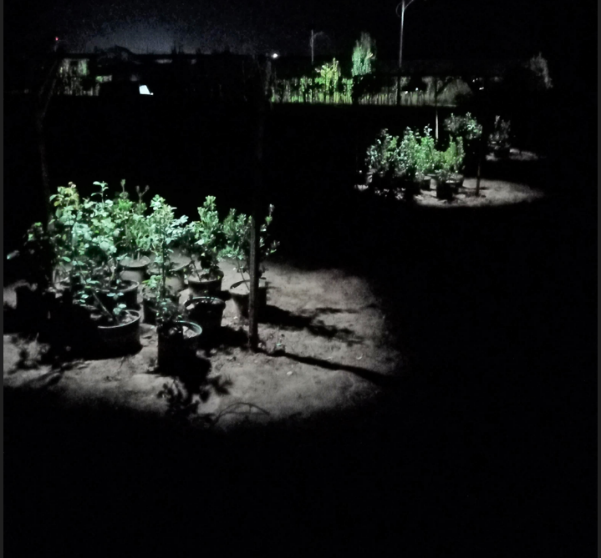 |
| --- | --- |

(b)

(a)

Fig.S1. Images of field experiment during day(a) and at night(b)

| 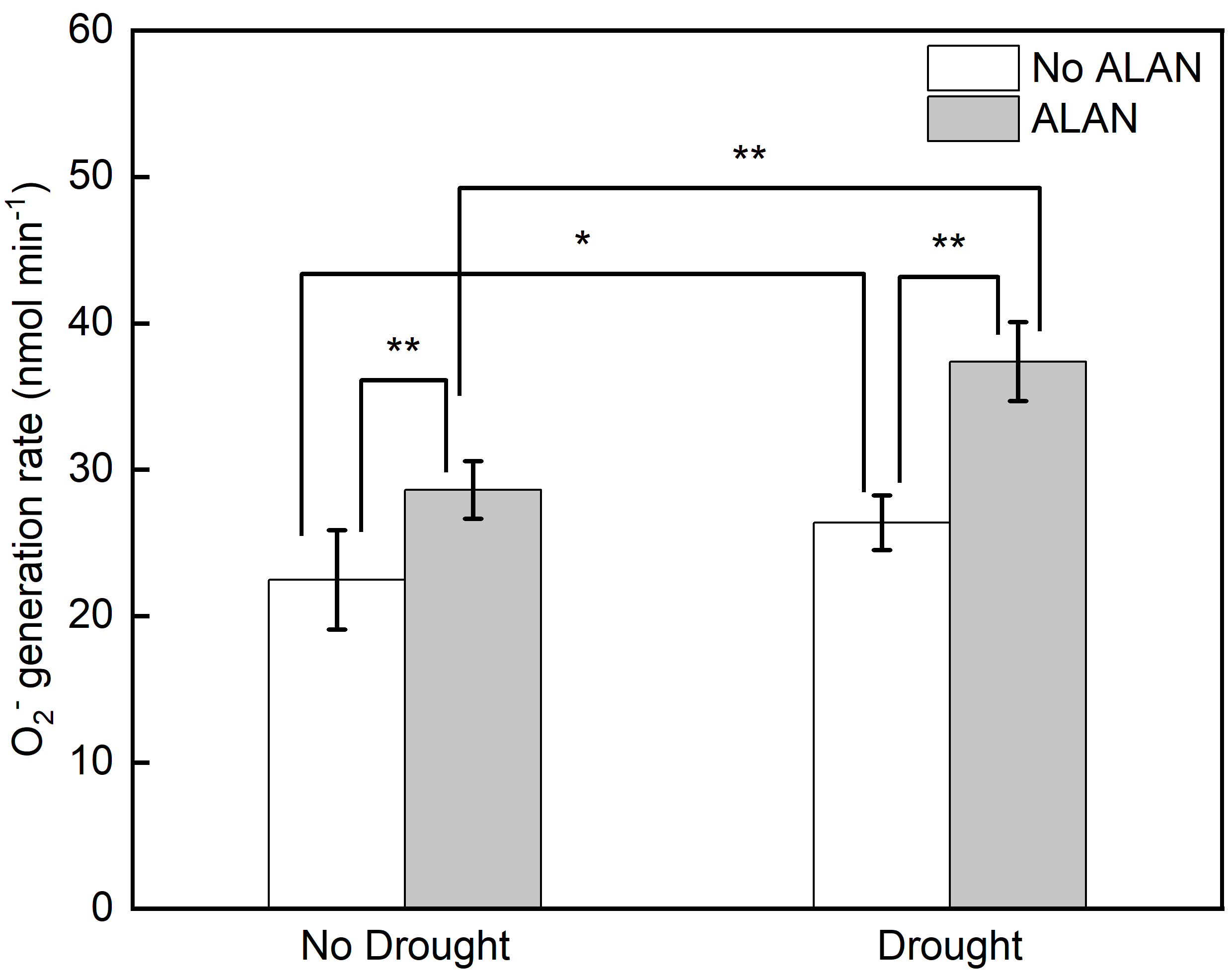  (a) | 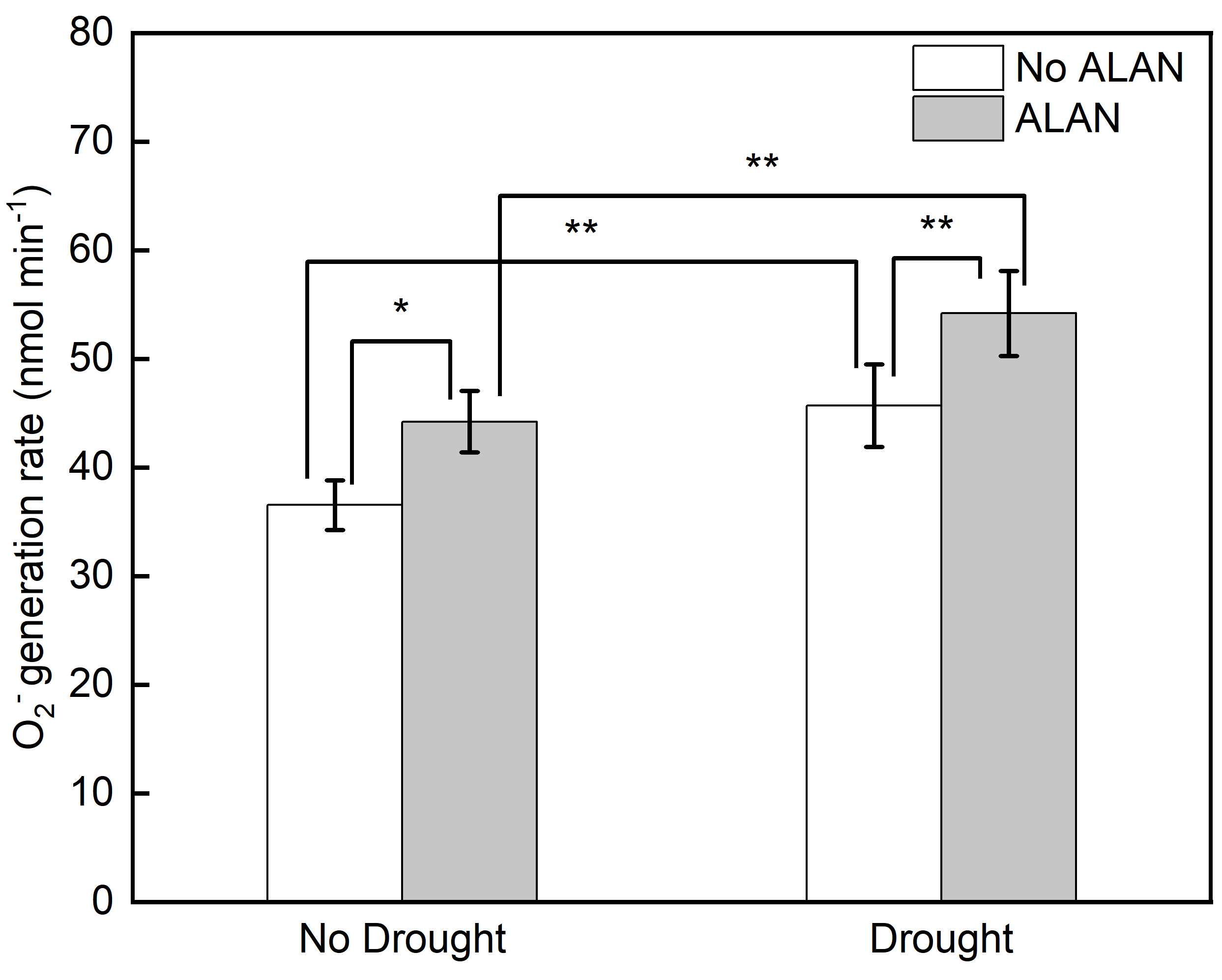  (b) |
| --- | --- |

Fig.S2. Effects of ALAN and drought on O_2_^-1^ generation rate of *E. japonicus* (a) and *R. hybrida* (b). Bars are means± SDs.

*Signifcant at p<0.05 and **Signifcant at p <0.001

Table S1 Test of between-subjects effects of ALAN and drought on O_2_^-1^ generation rate of *E. japonicus* and *R. hybrida*

| species | source of variation | O_2_^-1^ generation rate |
| --- | --- | --- |
| *E. japonicus* | ALAN | ** |
|  | Drought | ** |
|  | ALAN*Drought | * |
| *R. hybrida* | ALAN | ** |
|  | Drought | ** |
|  | ALAN*Drought | ns |

*Signifcant at p<0.05 and **Signifcant at p <0.001

Test methods for rate of production of O_2_^-^

Rate of production of O_2_^-^ was measured according to the method of Ke et al. (2002) with appropriate modification. About 1g fresh-frozen leaf tissue was homogenized with 3 mL of phosphate buffer. After the homogenate being centrifuged at 12000 × g for 20 min，0.5 ml supernatant,0.5 ml phosphate buffer and 1ml hydroxylamine hydrochloride(10 mmol /L) were mixed and was held at 25 ° C for 20 minutes. And then the mixture was added 2ml sulfanilic acid (17 mmol /L) and 2ml Alpha-naphthylamine (7 mmol /L). The sample was held at 25 ° C for 20 minutes, and then its light absorption at 530nm was measured by a spectrophotometer.

Ke, D.S., Wang, A.G., Sun, G.C., Dong, L.F., 2002. The effect of active oxygen on the activity of ACC synthase induced by exogenous IAA. Acta Botanica Sinica 44, 551-556.
